# Low-threshold optical bistability of graphene-wrapped dielectric composite

**DOI:** 10.1038/srep23354

**Published:** 2016-03-21

**Authors:** Yang Huang, Andrey E. Miroshnichenko, Lei Gao

**Affiliations:** 1 College of Physics, Optoelectronics and Energy of Soochow University, Collaborative Innovation Center of Suzhou Nano Science and Technology, and Jiangsu Key Laboratory of Thin Films, Soochow University, Suzhou 215006, China; 2 Nonlinear Physics Centre, The Australian National University, Canberra ACT 0200, Australia

## Abstract

We theoretically study the effective third-order nonlinear response and optical bistability of the 3D graphene based composite consisting of graphene wrapped dielectric nanoparticles embedded in dielectric host at terahertz frequencies. Taking into account the nonlinear conductivity of graphene, we derive the analytical expressions for the effective third-order nonlinear coefficient 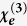 in weakly nonlinear limit. Moreover, for strong applied fields, the criterion for achieving optical bistability in such a graphene coated sphere, as well as the switching thresholds of optical bistability are discussed. We find that both 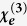 and optical bistability are strongly dependent on the Fermi energy of graphene and it is possible to achieve very low switching thresholds under the normal graphene dissipation. We further propose a scheme to study the transmittance of this nonlinear composite slab. These results reveal novel regime of the optical bistability of the transmittance of light. We show that this kind of graphene-wrapped composite, which has tunable and low threshold optical bistability, can be the best candidate for unique nonlinear optical materials.

Graphene is a two-dimensional hexagonal crystal carbon sheet with only single layer of atoms, which has recently attracted enormous interest for its outstanding optical properties^1^,^2^,^3^,^4^ and abundant potential applications in optoelectronic devices^5^,^6^,^7^,^8^,^9^,^10^,^11^,^12^,^13^, such as ultrafast optical modulator^3^, graphene photodetectors^11^ as well as graphene touch screens^12^. One novel feature of graphene is its controllable optical properties due to tunability of conductivity of graphene, which could give rise to some guidances on tunable optical sensor^14^, graphene metamaterials^15^,^16^,^17^, graphene plasmonics^18^,^19^,^20^,^21^,^22^,^23^,^24^,^25^, terahertz absorber^26^,^27^,^28^ and tunable Casimir force^29^.

One remarkable feature of graphene is its nonlinear properties which have already been considered both in theory and experiments^30^,^31^,^32^,^33^,^34^,^35^,^36^,^37^. Some exploitations have focused on the problems of bistability in graphene based structures^38^,^39^. Optical bistability (OB) existing in nonlinear optical systems shows the possibility to exhibit two different values of the transmitted light intensity for one input intensity^40^. It can give the optical structures the function to control two distinguishing stable transmission states with the history of the input light, which can be further used in switching, logic functions, modulation and so on. One challenge of OB in discipline is to achieve significant nonlinear interaction at ever smaller excitation powers and interaction volumes, while maintaining its tunability. Normally, strong OB can be realized in a media with high Kerr nonlinearity, where the material’s refractive index is efficiently modulated by the input light^41^,^42^. However, conventional Kerr-type nonlinear materials generally have very weak nonlinear response. In this connection, graphene is shown to has a large nonlinear Kerr index^32^,^38^, which result in its superior third-order nonlinear optical properties. Using graphene’s analogous Kerr nonlinearity on conductivity 
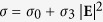
 32, OB has been widely investigated in one or two dimension structures^38^,^39^,^43^.

Although graphene is employed not only for planar geometries, it is possible now to create graphene wrapped objects experimentally^44^,^45^. To the best of our knowledge, few works are aimed at OB in 3D graphene based structure^46^. In the present work, we propose the 3D graphene based composite in which graphene coated dielectric spheres are randomly embedded in the linear host medium. Within the quasi-static approximation, we derive and theoretically study its effective third-order nonlinear coefficient, which is an important parameter in the nonlinear response of the composite. In addition, OB is reported in this novel 3D graphene wrapped spheres with our proposed theoretical method. By rigorous derivation, we show that the composite system could provide more parameter space to achieve low threshold OB with a lower Fermi-level energy of graphene at terahertz frequency, compared to the low-dimensional structures. Furthermore, we investigate the optical transmittance of the nonlinear composite slab. Zero transmittance is found at all incident angles with subwavelength thickness of the linear composite system, and unusual nonlinear OB behavior is reported in which the transmittance can be switched from almost zero to a very high level at low incident field strength. This proposed 3D graphene based nonlinear composite might provide a new thought to the design of tunable optical devices.

## Results

### Analytical Derivation

**Figure 1:**
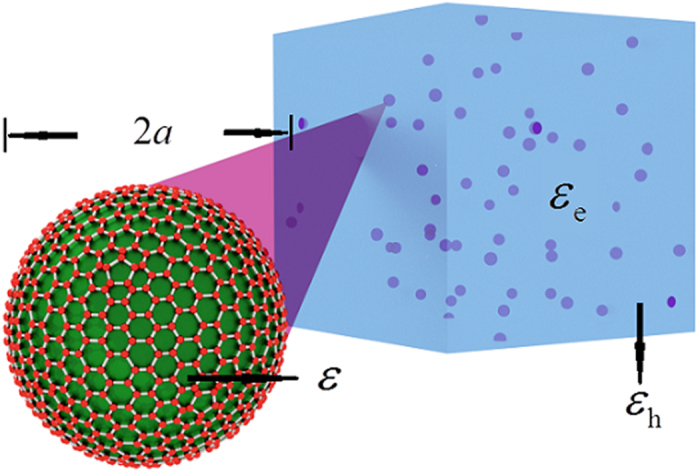
Schematic diagram of the graphene wrapped composite in which the dielectric spherical inclusions are coated by graphene layers.

We consider a random composite in which graphene wrapped spherical dielectric nanoparticles with radii *a* are randomly embedded in the linear dielectric host as shown in Fig. 1. On the experimental side, the graphene wrapped sphere can be achieved by using electrostatic self-assembly, in which process the spherical dielectric core is initially modified to acquire a positive charge and then co-assemble with the negatively charged graphene sheet. Based on this method, many graphene-wrapped nanoparticles have been fabricated^44^,^45^,^47^, even for some more complicated particles with hollow core^48^,^49^. One specific example was in the work done by Zhao’s group^50^, who fabricated graphene wrapped mesoporous Silica nanoparticles with the total radius around ~50 nm which is very similar to our model. Besides the electrostatic self-assembly, graphene wrapped dielectric particles can be fabricated in an emulsification process^51^ as well. Though experimental deviation from an idealized spherical graphene coating, as well as surface roughness, can’t be avoided, the above experimental evidences underscore the relevance of the geometry beyond a theoretical perspective. Therefore, in the theoretical model^22^,^34^,^52^, the dielectric constants of the dielectric spherical particles and host are given as *ε* and *ε*_h_, respectively. Assuming the electric displacement vector **D**_*n*_ inside (*n* = c) and outside (*n* = h) the sphere have linear relation with the electric fields **E**_*n*_, so that **D**_*n*_ = *ε*_*n*_**E**_*n*_. In the case when *a* is far less than the wavelength of the incident light, we can adopt the so-called the quasi-static approximation. Hence the electric potentials both inside and outside the spherical particles would satisfy the Laplace equation: 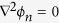. In the case of linearly polarized plane monochromatic electromagnetic wave incidence (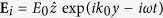), the general solutions of the Laplace equations can be derived to be,


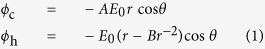


Here, the electric potential inside the particles includes the incident part and induced one, i.e., 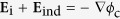. As to *ϕ*_h_, we have 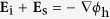 in which **E**_s_ is the scattering field in the host. *E*_0_ is the amplitude of the external applied field and *A*, *B* are the unknown coefficients to be determined with the appropriate boundary conditions. For this monolayer graphene coated dielectric sphere in which the graphene layer is considered as an extremely thin conducting shell with conductivity *σ*, we employ the non source-free boundary conditions^34^,


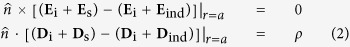


where *ρ* is the surface density of charge which has the relation 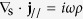 with surface density of linear current 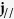 at the frequency *ω*. Here, the operator ∇_s_ stands for surface divergence and 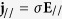 with the tangential field component 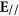 of the induced field and the surface conductivity *σ*.

Solving the above equations, one yields the unknown coefficients,


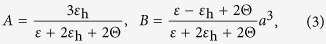


where 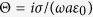 and *B* is dipole polarizability of the coated sphere. According to the Clausius-Mossotti relation, we can get the effective dielectric coefficient^53^,


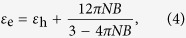


in which *N* is the numbers of spherical particles per volume. Substituting *B* into the Eq. (4), we have


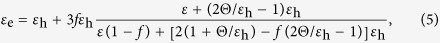


where 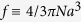 is the volume fraction of the particles.

In the weakly nonlinear limit, to derive the third-order coefficient of the composite, it is convenient to introduce the macroscopic effective linear dielectric constant 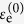 and the effective third-order nonlinear optical susceptibility 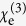 as follows^54^,^55^:


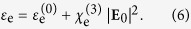


Next, we rewrite Eq. (5) in the form of Eq. (6) with nonlinear surface conductivity of the monolayer graphene and derive explicit expression for 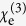 by comparing them. To model the surface conductivity of the graphene, we introduce the simplified version within the random-phase approximation,


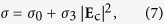


in which the linear term *σ*_0_ = *σ*_intra_ + *σ*_inter_, *σ*_intra_ and *σ*_inter_ are the intraband and interband terms which have the following forms^39^,


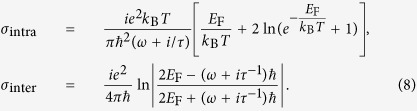


In the above equation, 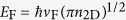 is the Fermi energy which can be electrically controlled by an applied gate voltage due to the strong dependence of the carrier density *n*_2D_ on the gate voltage, *τ* is the electron-phonon relaxation time, and *T* is the temperature in K. *e*, 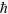, *k*_B_ and 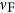 are the electron charge, reduced Planck constant, Boltzmann constant, and the Fermi velocity of electrons respectively. Here we would like to mention that the conductive response of the graphene thin shell is approximated by its planar equivalent. Such a treatment has been adopted in recent theoretical works^22^,^34^,^52^. Generally, zone folding for planar graphene quantizes the allowable electronic momenta and hence modifies the linear energy dispersion. However, these perturbation incur only negligible changes to the conductive response provided the inverse circumference remains small relative to the Fermi momentum 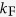, i.e., provided 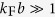
22. Moreover, in this work, we consider the dielectric core having a radius ~100 nm, which is much larger than the thickness of graphene. Hence the graphene coating can be characterized well as a two-dimension homogenized conducting film where the non-spherical elements and microscopic details are neglected^4^. The third-order nonlinear surface conductivity *σ*_3_ can be expressed as^32^


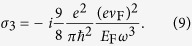


In THz frequencies, the nonlinear term of the surface conductivity of graphene is much weaker, i.e., 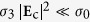. Thus, using Taylor expansion method, we can approximate Eq. (5) as


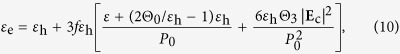


where 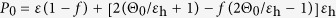, 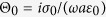 and 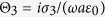.

On the other hand, in the composite system, for the local electric field inside the sphere **E**_c_ and outside the sphere **E**_h_ there is a relation


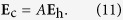


To derive the relationship between the external field **E**_0_ light upon the composite and **E**_c_, one may introduce an average field theory for the composite,


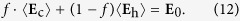


Therefore, we can finally derive the following relation


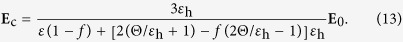


In the weakly nonlinear limit, substituting Eq. (7) into Eq. (13) and keeping terms only to first order in *σ*_3_, we obtain


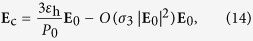


It is sufficient to only retain the first term in Eq. (14) and substitute for **E**_c_ in Eq. (10),


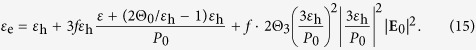


By comparing Eq. (15) and (6), we can obtain 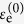 and 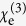 as


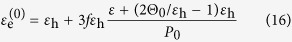


and


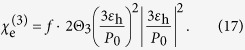


In what follows, we would like to check the strong field case. Generally, Eq. (13) is the nonlinear equation for the local field **E**_c_ inside the particles as the function of **E**_0_. Taking the square of modulus of Eq. (13) one yields,


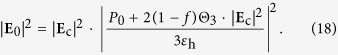


For frequencies 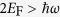, 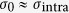 because the interband transitions in graphene are forbidden by the Pauli exclusion principle. And 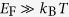 in the room temperature (*T* = 300 k), hence the linear part of the surface conductivity in Eq. (8) can be a simplified version as,


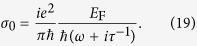


In the case that *ε*_h_ and *ε* are pure dielectric and have no dissipation, we can alternatively rewrite Eq. (18) as


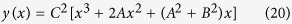


by defining new dimensionless variables 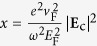 and 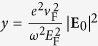, where


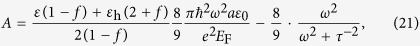



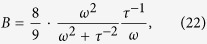



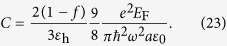


In order for Eq. (20) to achieve OB response, the following additional inequalities should be satisfied^56^,


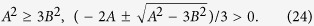


Thus the positions of the local minimum (maximum) 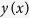 are at 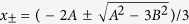, and the switching-up/down threshold of OB is 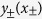.

### Effective Third-order Nonlinear Coefficient

**Figure 2:**
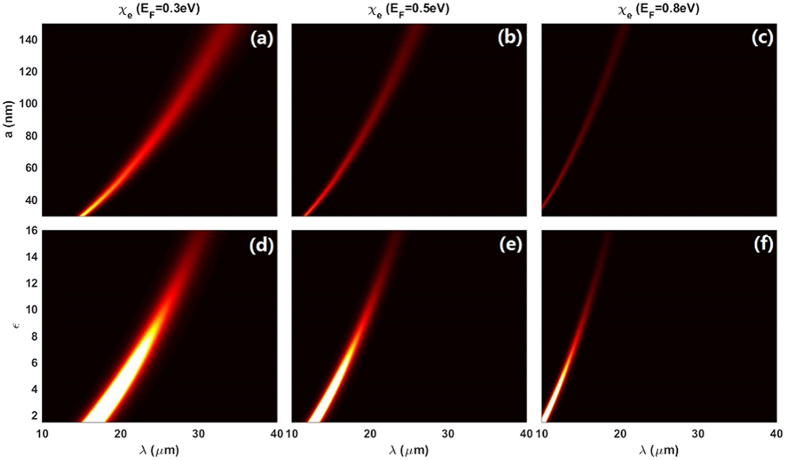
Upper panel: the effective third order nonlinear coefficient 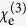 as the functions of external incident wavelength *λ* and the particle radius *a* with *E*_F_ being (**a**) 0.3 eV, (**b**) 0.5 eV and (**c**) 0.8 eV respectively. The dielectric constant of the dielectric particle is chosen as *ε* = 12.25. Lower panel: 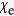 as the functions of *λ* and *ε* with *E*_F_ being (**d**) 0.3 eV, (**e**) 0.5 eV and (**f**) 0.8 eV respectively. The radius of the dielectric particle *a* is 100 nm. Other parameters are: 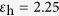, 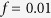, and 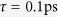.

In Fig. 2, we plot 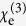 as functions of several variables based on Eq. (17). Within the parameters we choose, large Fermi energy level will generally lead to small magnitude of the resonant nonlinearity enhancement peak, accompanied by the blue-shift. In addition, increasing the radius *a* and permittivity *ε* of the particles could further reduce the magnitude of the resonant peak. However, in contrast to *E*_F_, increasing the radius *a* and permittivity *ε* of the particles will result in the peak of 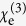 red-shifted. As a result, in order to get large nonlinear response of the composite, one may either reduce the geometric/physical parameters, i.e., *a* and *ε*, or decrease the graphene related variable *E*_F_. Note that, for larger 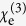 we concentrate on the peak value in spectra, we do not compare 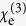 as the function of *a*, *ε* and *E*_F_ at a specific *λ*. This conclusion may be derived directly from analysis of Eq. (17). We would like to mention that the wavelength range chosen here could keep the quasistatic approximation valid, and smaller *a* and *ε* will go a further step to verify this validity.

### Optical Bistability of Near –Field

The effective third order nonlinear coefficient 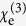 indicates how strong nonlinearity the composite may possess. Normally, it is important to find a high 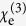 for composites if one wants to achieve dramatic OB with low switching threshold. On the other hand, OB only occur when the parameters satisfy some specific conditions as shown in Eq. (24). Here we derive the OB criterion for the composite as


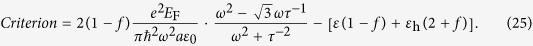


**Figure 3:**
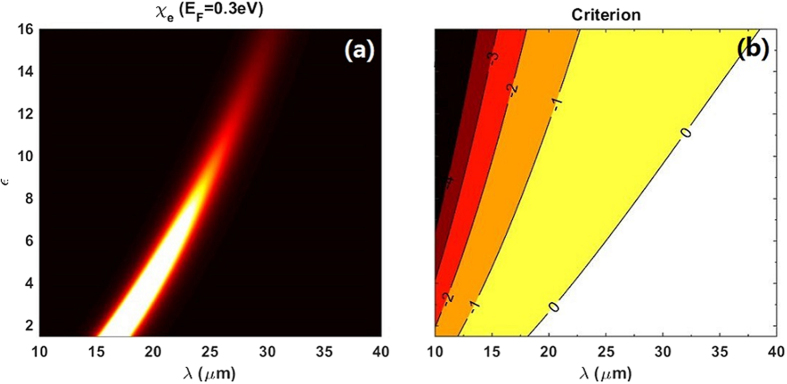
(**a**) Same as shown in Fig. 2(d). (**b**) Criterion as the functions of the external incident wavelength *λ* and the particle radius *a*. Other parameters are the same as (**a**). Solid lines with numbers are contours of the theoretical results of Eq. (25). Only the parameters laid on the regions where the contours are larger than 0 can give rise to optical bistability in the composite.

**Figure 4:**
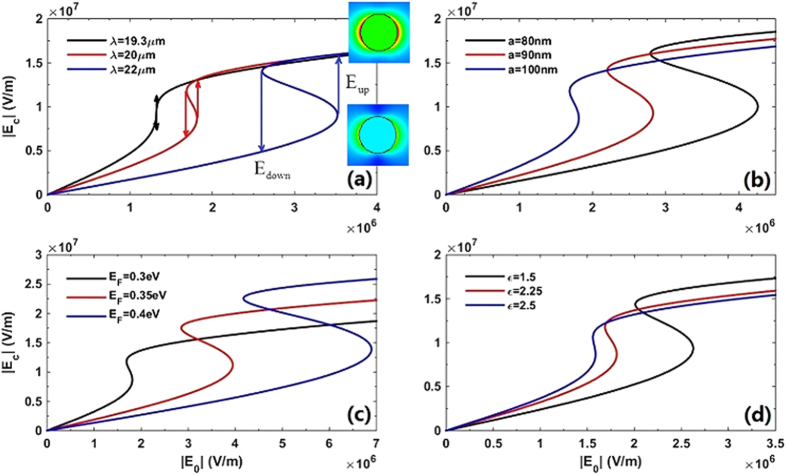
Dependence of the local electric field intensity 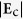 on the external incident field 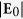 with various (**a**) *λ*, (**b**) *a*, (**c**) *E*_F_ and (**d**) *ε*, respectively. Parameters are *E*_F_ = 0.3 eV, *a* = 100 nm, *ε* = 2.25 for (**a**), 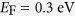, *λ* = 20 μm, 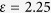 for (**b**), *a* = 100 nm, *λ* = 20 μm, *ε* = 2.25 for (**c**) and *a* = 100 nm, *λ* = 20 μm, 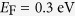 for (**d**). Other parameters are *ε*_h_ = 2.25, 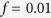 and 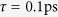. The inserts in (**a**) are the near-field distributions of inclusions before and after 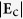 dumping at 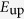 respectively.

Only for the condition that *Criterion* > 0, this composite could exhibits OB. Viewing Fig. 3, two conclusions can be made, one is that OB could not be achieved where 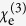 reaches its maximal value with the present parameters. The other one is that the critical boundary for OB in composite is more close to high 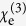 at lower *ε* and *λ* within our interested parameters regions. To achieve low switching threshold for OB, we choose *ε* = 2.25 and *λ*=19.3 μm as our primary parameters in Fig. 4(a) because they just lie on the critical boundary for OB and have a corresponding high value of 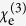. The solid back line shows this critical condition that the switching-up and switching-down thresholds 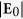 are the same, and their switching thresholds are very low, almost one order of magnitude smaller than the enhanced local field 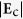. To make comparison, another two curves with longer wavelength (*λ* = 20 μm and *λ* = 22 μm) are plotted in the same figure. The longer the *λ* is, the lower value of 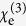, hence the higher switching thresholds. With the explicit expressions for 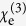 and criterion of OB, it is easy to find appropriate parameters to achieving low switching thresholds for OB.

Considering the relation between 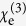 and the switching threshold of OB, the dependence of the switching thresholds on *λ*, *a*, *ε* and *E*_F_ should be the same as that of 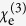. To one’s interest, the parameters space for achieving OB always lie on the right side of the maxima line of 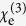 in Fig. 3. That means the switching thresholds are monotonically increasing (or decreasing) by the changing of these variables (*λ*, *a*, *ε* and *E*_F_), which are shown in Fig. 4 (b–d) and can be indicated by Eq. (17) as well. In details, we found that longer *λ* and higher *E*_F_ would lead to higher switching thresholds. On the contrary, larger *ε* and *a* give rise to lower switching thresholds. Below, we will further demonstrate that the switching thresholds always vary monotonically with these variables.

### Role of the Relaxation Time

**Figure 5:**
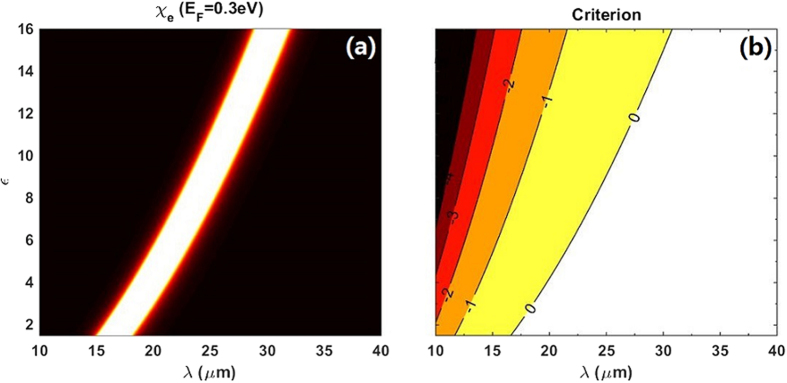
The same as Fig. 3 but with *τ* = 1 ps.

**Figure 6:**
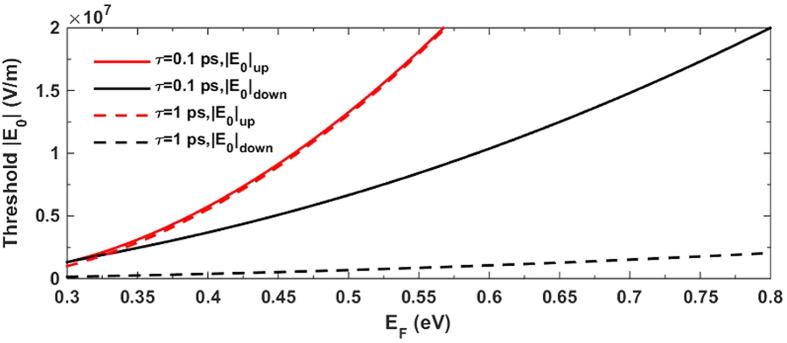
The dependencies of the switch-up and switch-down threshold electric fields |E_0_| on the Fermi energy *E*_F_ of the graphene with relaxation time *τ* = 0.1 ps (solid lines) and *τ* = 0.1 ps (dashed lines). The other parameters are 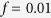, *a* = 100 nm, *ε* = 2.25, *ε*_h_ = 2.25 and *λ* = 19.3 μm.

In the discussion above, we employ the relaxation time of carriers as *τ* = 0.1 ps in our calculations. It is interesting to see how the relaxation time *τ* influence the third-order nonlinear coefficients and optical bistability. As an example, we replot Fig. 3 in Fig. 5 but with different *τ*. By comparing these two figures, we found that the magnitude of the optical nonlinearity peaks in our interesting spectra is generally increased with the increase of *τ*, especially for high *ε*. And the peak positions of 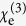 are slightly red-shifted as well. This is easy to understand because long relaxation time means low dissipation in graphene, hence resulting in small imaginary part of 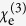 in Eq. (17). In addition, the parameters space for OB has been broadened when *τ* becomes larger, and the critical boundary (Criterion = 0) goes closer to the maxima line of 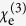. Based on the analytical solutions of Eq. (20), we plot the switching-up and switching-down threshold electric fields 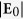 in the function of *E*_F_ with different *τ* in Fig. 6. As expected, the switching threshold 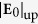 and 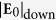 are both lower on the condition of τ = 1 ps than that of τ = 0.1 ps. However, the magnitudes are quite different. The switching-down threshold 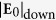 is much more sensitive to *τ*, and it decreases dramatically with longer *τ* especially for high *E*_F_ region. In contrast, 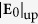 is less sensitive to the change of *τ* and shows slight decreasing. Figure 6 also confirms our conclusion that both 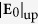 and 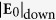 go higher with the higher *E*_F_. In other words, if we further reduce the Fermi energy *E*_F_, the switching thresholds will be even lower. It should be noted that the parameters for the solid lines in Fig. 6 are chosen right on the critical boundary for OB when *E*_F_ = 0.3 eV. That is why these two solid (red and black) lines finally cover each other at the leftmost. In this case, further reducing *E*_F_ will go beyond the condition for OB. But in the dashed line case, there still exists the region for decreasing *E*_F_, hence achieving lower switching thresholds.

**Figure 7:**
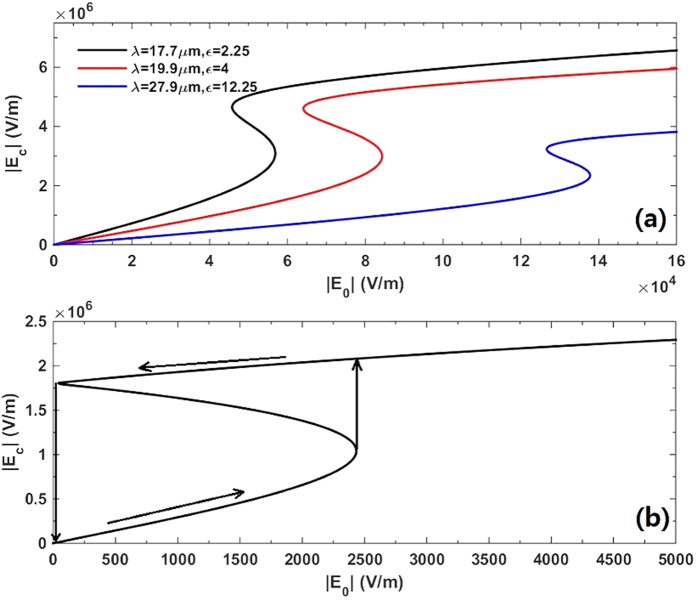
Dependence of the local electric field intensity 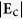 on the external incident field 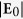 with (**a**) 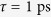 and (**b**) 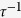 being 0. Where 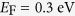, 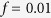, *a* = 100 nm, *ε*_h_ = 2.25 and *λ* = 17.5 μm. The permittivity of the sphere 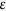 is 2.25 in (**b**).

Increasing *τ*, on one hand, will reduce the switching thresholds especially for 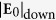. On the other hand, it provides us more parameter space to realize OB with low switching thresholds. In Fig. 5(b) we plot the results for three points along the critical boundary and plot their OB curves in Fig. 7(a). It clearly shows that they have very low switching thresholds on the order of 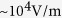 which are two order of magnitude smaller than these in Fig. 4. One can find a lot of other points in Fig. 5(b) which could provide very low switching thresholds with different parameters, in order to satisfy the realistic requirement in experiments.

Next, we want to discuss about one specific case in which 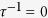. The situation can be quite different and interesting if we totally neglect the *τ*^−1^ term in the linear part of the surface conductivity. Therefore Eq. (19) would reduce to 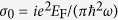, and other derivations would be rewritten accordingly. The criterion condition for OB is reduced to


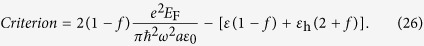


It is interesting to note that Eq. (26) is the same as *P*_0_ in the non-dissipation case. As a consequence, 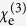 in Eq. (17) apparently reaches to the maxima when the criterion equation equals zero. This means that one can not achieve OB with the maximal effective nonlinear coefficient 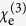. However, there is still room for OB with strong nonlinearity by slightly removing the wavelength *λ* into a bit higher value so that the OB criterion can be satisfied. Once the τ^−1^ is neglected hence *σ*_0_ is pure imaginary, the switching thresholds 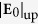 and 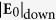 would be much more simplified and could be written as,


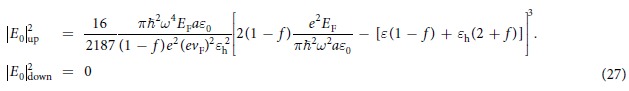


We are surprised to see the switching-down threshold is theoretically zero with any parameters only if the OB criterion is satisfied. After 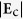 jumping to a high level when the external electric field intensity 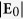 reaches to 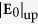, further decreasing 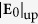 could only slightly reduce 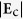. 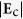 could keep a very high level and would not drop down to zero until 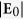 is completely zero [see Fig. 7(b)]. That means that the local field can be very high even the composite is incident by a light with extremely low field intensity. In realistic situations, the relaxation time *τ* should be included and any small *τ*^−1^ will lead to 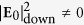. 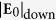 becomes large with the increase of *τ*^−1^. In addition, any amount of *τ*^−1^ will lead to the criterion boundary deviating from the maxima line of 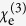 and always laying on the right side of it. That’s why the switching thresholds always vary monotonically with these variables.

Up to now, we demonstrate that, in order to reduce the switching thresholds one can either increase the particle radius *a* and permittivity *ε* of the particle, or reduce the external wavelength *λ* and Fermi energy *E*_F_. The introduction of graphene provides us a new freedom to operate the OB in the composite. Once the structure parameters, i.e., *a* and *ε* are fixed, we can still change the OB profile by varying *E*_F_ as well as the conventional variable *λ*. In principle, we can achieve very low switching threshold OB at both low and high THz frequencies. Unlike the graphene-dielectric multilayer structures^38^,^39^, the presented composite can exhibit OB at high THz frequencies with very low *E*_F_, as well as low switching thresholds.

### Transmittance of the Composite Slab

**Figure 8:**
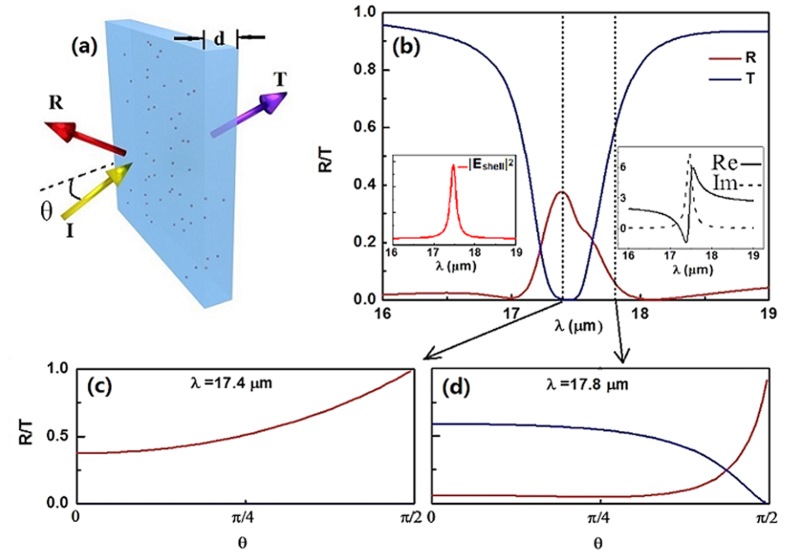
Transmission spectra of the slab composed of linear composite material with thickness d = 5 μm. (**a**) The schematic diagram of the light scattering by the slab. (**b**) The reflectance (R) and transmittance (T) as the function of incident wavelength 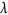 at normal incidence (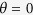). R and T versus the incident angle 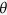 at (**c**) *λ* = 17.4 μm and (**d**) *λ*=17.8 μm. The insert in (**b**): (left) 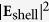 as the function of incident wavelength; (right) the real (solid) and imaginary (dashed) parts of 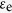.

In order to verify the properties of this composite material for more practical purpose, we next study the transmittance and reflectance of the subwavelength slab consisting of this kind of composite. Without loss of generality, we employ the field-dependent effective permittivity *ε*_e_ similar as Eq. (5) to describe the nonlinear optical response of the composite slab. The reflectance and transmittance are written as follows,


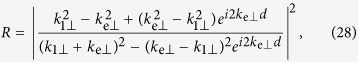



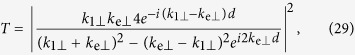


where 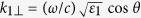 and 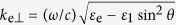 are the normal components of the wave vectors in the surrounding medium with permittivity *ε*_1_ and slab with field-dependent permittivity *ε*_e_ respectively. *θ* and *d* are the incident angle and thickness of the composite slab [see Fig. 8]. First, we consider the transmission spectra from the linear composite slab. For the normal incidence, Fig. 8(b) shows a completely zero dip of transmittance at the surface plasmon resonant wavelength *λ* = 17.4 μm in the spectrum, and high transmittance dominates in the spectrum for the non-resonant incident wavelengths. Actually, this surface plasmon resonant wavelength can be well determined by our effective permittivity of the composite system Eq. (5) [or Eq. (15)], i.e., the imaginary part of the denominator in Eq. (5) equals to zero. At the surface plasmon resonant wavelength, the local field within graphene thin shell will be largely enhanced due to the confining optical mode on the graphene shell layer [see the left insert of Fig. 8(b)], resulting in zero transmission at *λ*=17.4 μm. Therefore although the composite slab is only 5 μm thin the light can not go through the slab, not only at normal incidence but also at all angles [see Fig. 8(c)]. On the contrary, transmittance would be very high due to the low inherent dissipation of graphene off the surface resonant wavelengths [see Fig. 8(d)].

**Figure 9:**
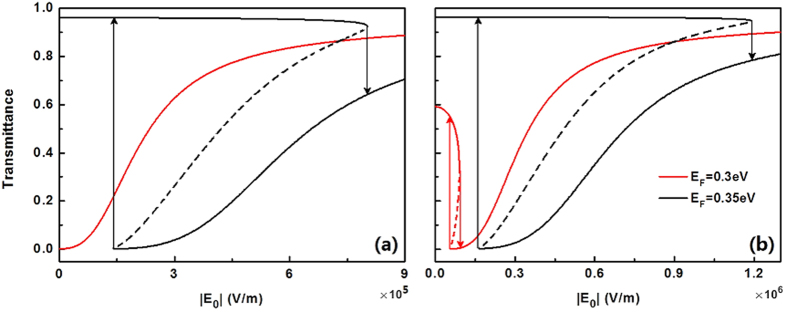
Dependence of transmittance on external incident field 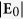 at different Fermi-level of graphene at (**a**) *λ* = 17.4 μm and (**b**) *λ* = 17.8 μm. Other parameters have the same values as these in Fig. 8.

The enhancement of local fields within the nonlinear graphene thin shell at the surface plasmon resonant wavelength represent an ideal condition to boost the nonlinear effects in the graphene thin shell. Next we would like to check the transmittance spectra when the high field is applied. We find that it still has the opportunity to go through the composite slab and has a very high transmittance for *E*_F_ = 0.3 eV at the resonant wavelength *λ* = 17.4 μm [see the red line in Fig. 9(a)]. In detail, at the beginning, with increasing 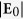, the transmittance of the slab increases slowly, after that however it goes high rapidly until 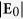 reaches to ∼5 × 10^5^ V/m. With the present parameters, the red line does not show a bistable curve. Once we adjust the Fermi energy *E*_F_ = 0.35 eV, the transmittance curve indicate OB. In contrast to the red line, the transmittance of OB (black line) is high at low 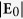 and jumps to a low level at a high value of 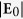. When decreasing 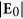, it first goes low which is almost zero (*T* ≈ 0) but finally jumps to a dramatically high transmittance (*T* = 0.96) at low incident intensity. And we find its switching thresholds are not very high, only on the level of ∼10^5^ V/m. This novel properties of transmittance can be used as tunable optical switches and sensors. For comparison, we plot the R/T curves off the resonant wavelength *λ* = 17.8 μm in Fig. 9(b). For 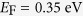, low switching-threshold OB is predicted, but it is still higher than that at 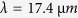 under the same condition [see Fig. 9 (a,b)]. Again, near the surface plasmon resonant wavelength, much optical energy is confined to the nonlinear graphene thin layer, resulting in lower optical threshold.

In the end, some comments are in order. At first, we assume that the model is within the quasi-static approximation since the radius of particle is far less than the incident wavelength. Note that the radius is less than100 nm, while the incident wavelength is around 10 μm, hence the quasi-static approximation is safely satisfied. Secondly, to describe the surface conductivity of graphene, we introduce the random-phase approximation, in which the surface conductivity has a simplified form. And this conductivity expression further reduces to a simpler one [see Eq. (19)] when we choose the condition 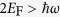. Finally, we neglect the influence of random fluctuation in potential along the surface of graphene in this work. Random fluctuations in potential will result in the fluctuations in charge carrier density and play a role in the surface conductivity of the graphene. Actually, the fluctuations in charge density and the corresponding electrostatic potential were found to affect the plasmon dispersion and damping of graphene plasmonics^57^. Since our main results are dependent on the assumed surface conductivity, therefore, it will be expected to influence the reliability of the proposed devices. The random fluctuations in potential may result in qualitatively the graphene’s surface conductivity being nonlocal and graded. For the quantitative calculations, one needs to adopt the first principle to derive the disorder-(random fluctuations) dependent graphene’s surface conductivity. For this part, it requires separate and exhaustive study, and will be reported elsewhere.

## Conclusions

To conclude, we propose a new nonlinear composite composed of graphene-wrapped nanoparticles embedded in dielectric host. In the quasi-static limit, we derive the effective third-order nonlinear coefficient and study the optical bistability (OB) of the composite at terahertz frequencies. We found that by decreasing the Fermi energy of the graphene layer, as well as the radius and permittivity of the inclusions, one could effectively increase the third-order nonlinear response. With rigorous derivation, the conditions for achieving optical bistability is presented in addition with the switching thresholds of OB. It is shown that the switching thresholds are highly dependent on the Fermi energy of graphene, therefore, it provides a new degree of freedom to control the inside local field with input one. Moreover, tunable conventional variables (radius or permittivity of the inclusions) of the nonlinear composite could be used to reduce the switching thresholds in the meantime keeping a low Fermi energy level, which might be practicable for experiments. In general, longer *λ* and higher *E*_F_ would lead to higher switching thresholds. And larger *ε* and *a* give rise to lower switching thresholds. Furthermore, the influence of the relaxation time of graphene is discussed. It is interesting to note that when the relaxation time is infinitely long, the switching-down threshold is down to zero which indicate the state that extremely small input could lead to large enhanced local field inside the composite. Finally, we study the optical reflectance and transmittance of the subwavelength slab consisting of such graphene-based nonlinear composite. Complete zero transmittance is found at all incident angles, and the transmittance also show giant OB with the variation of incident field intensity which can be tuned by the Fermi energy of the graphene. Graphene optical bistable devices appear to be particularly promising and could open a new possibility of all-optical switching, optical transistor, optical logic and memory.

## Additional Information

**How to cite this article**: Huang, Y. *et al.* Low-threshold optical bistability of graphene-wrapped dielectric composite. *Sci. Rep.* **6**, 23354; doi: 10.1038/srep23354 (2016).
